# Hydrogels as scaffolds and delivery systems to enhance axonal regeneration after injuries

**DOI:** 10.3389/fncel.2015.00013

**Published:** 2015-02-17

**Authors:** Oscar A. Carballo-Molina, Iván Velasco

**Affiliations:** Instituto de Fisiología Celular-Neurociencias, Universidad Nacional Autónoma de MéxicoMexico, D.F., Mexico

**Keywords:** axotomy, growth factors, injury response, grafting, surgical intervention

## Abstract

Damage caused to neural tissue by disease or injury frequently produces a discontinuity in the nervous system (NS). Such damage generates diverse alterations that are commonly permanent, due to the limited regeneration capacity of the adult NS, particularly the Central Nervous System (CNS). The cellular reaction to noxious stimulus leads to several events such as the formation of glial and fibrous scars, which inhibit axonal regeneration in both the CNS and the Peripheral Nervous System (PNS). Although in the PNS there is some degree of nerve regeneration, it is common that the growing axons reinnervate incorrect areas, causing mismatches. Providing a permissive substrate for axonal regeneration in combination with delivery systems for the release of molecules, which enhances axonal growth, could increase regeneration and the recovery of functions in the CNS or the PNS. Currently, there are no effective vehicles to supply growth factors or cells to the damaged/diseased NS. Hydrogels are polymers that are biodegradable, biocompatible and have the capacity to deliver a large range of molecules *in situ*. The inclusion of cultured neural cells into hydrogels forming three-dimensional structures allows the formation of synapses and neuronal survival. There is also evidence showing that hydrogels constitute an amenable substrate for axonal growth of endogenous or grafted cells, overcoming the presence of axonal regeneration inhibitory molecules, in both the CNS and PNS. Recent experiments suggest that hydrogels can carry and deliver several proteins relevant for improving neuronal survival and axonal growth. Although the use of hydrogels is appealing, its effectiveness is still a matter of discussion, and more results are needed to achieve consistent recovery using different parameters. This review also discusses areas of opportunity where hydrogels can be applied, in order to promote axonal regeneration of the NS.

## Introduction

The nervous system (NS) is responsible for the interaction between organisms and their environment; it confers the ability to respond to external stimuli. However, when an injury occurs in this system, such ability is impaired. Understanding fundamental mechanisms involved in the response to damage might be used to design therapeutic interventions aimed to promote functional recovery. Axonal regeneration capacity is very limited in the Central Nervous System (CNS; Gurgo et al., 2002; Case and Tessier-Lavigne, 2005). Although the Peripheral Nervous System (PNS) is able to grow axons after a nerve injury, the lost function is not always restored, because the regenerated axons are unable to reinnervate areas previously connected by them (Johnson et al., 2005). This review describes first the elements that impede axonal regeneration following injury in CNS and PNS, and later discuss how hydrogels might attenuate the inhibitory elements for axonal regeneration in both systems.

## Nervous system response to injury and its role in axonal growth inhibition

An injury in the NS could imply a loss of tissue, interrupted communication caused by damage of synaptic contacts or disrupted information flow between cell soma and axons. Different events occur after NS damage, depending on several factors, such as the type of injury. Many of these events are responsible for the inhibitory environment during axonal regeneration. We next describe the differential responses to lesion of CNS and PNS.

### CNS

#### Glial and fibrous scar formation

**Figure 1:**
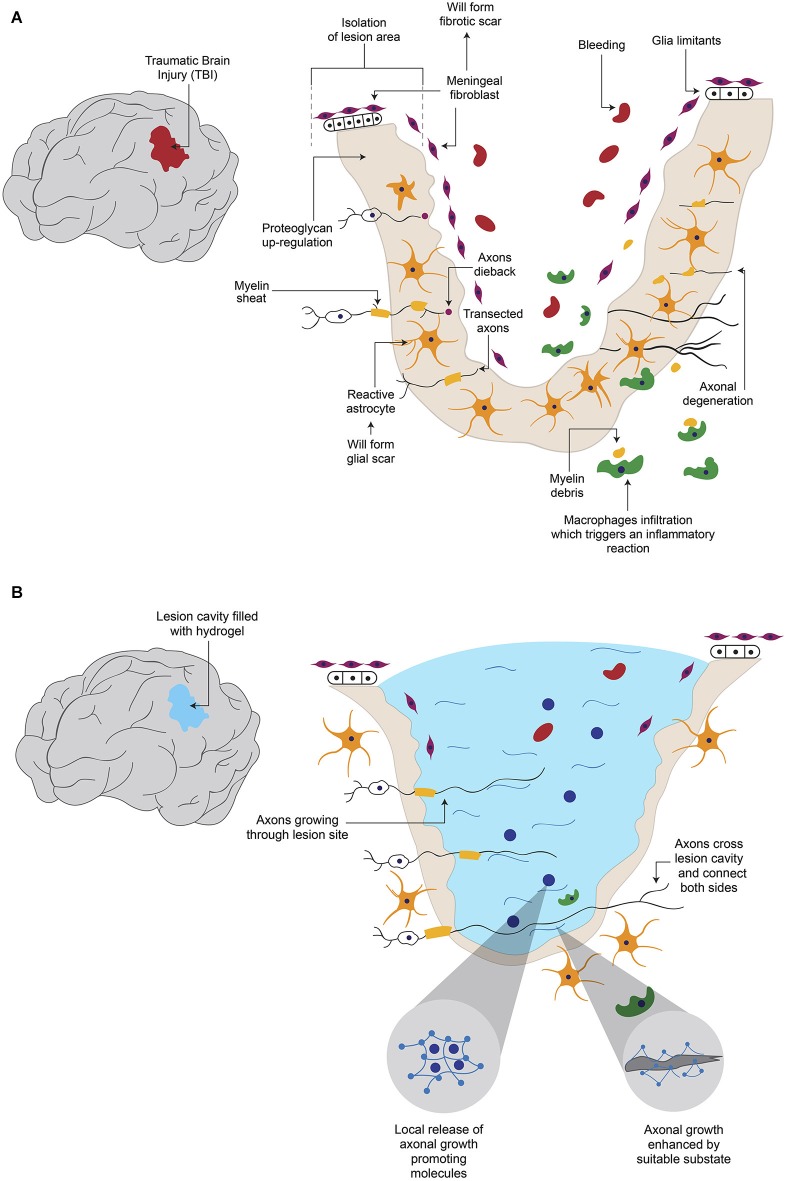
**Hydrogel promotes axonal regeneration after Traumatic Brain Injury (TBI). (A)** After a TBI, which causes axonal degeneration, inhibitory elements for axonal regeneration such as the formation of a cavity in the tissue, fibrotic and glial scars and reactive astroglia, are present. **(B)** When the newly formed cavity is filled with hydrogel, it provides a suitable substrate for axonal growth, in addition to the possibility to be combined with molecules that are released from hydrogel to enhance regeneration.

The disruption of the blood brain barrier (BBB) after damage allows the infiltration of blood proteins to the CNS, which triggers an inflammatory reaction (Kawano et al., 2012). White cells and macrophages enter through the lesion site and migrate to the surrounding neural tissue, releasing various cytokines and chemokines (Merrill and Benveniste, 1996; Donnelly and Popovich, 2008). These events lead to the activation of astrocytes, microglia and oligodendrocyte progenitor cells, to form the glial scar around the lesion site (Shearer and Fawcett, 2001; Kawano et al., 2012; Figure 1A). These activated cells release different molecules involved in inflammation, BBB restoration and neuroprotection (Yiu and He, 2006; Rolls et al., 2009). Glial scar isolates the damage area from adjacent tissue (Figure 1A); this contributes to maintaining homeostatic functions as ion and fluids balances, production of pro- and anti-inflammatory molecules, secretion of growth factors and free radicals elimination (Yiu and He, 2006; Rolls et al., 2009). In addition, a fibrotic scar (Figure 1A), which is produced by the intrusion of fibroblasts from the damaged meninges and that release extracellular matrix (ECM) proteins such as type IV Collagen, Fibronectin and Laminin, also forms around the site of lesion (Kawano et al., 2012). Moreover, fibroblasts and astrocytes cooperate to establish a continuous basal lamina around the glial scar (Mathewson and Berry, 1985; Shearer and Fawcett, 2001). The barrier formed by the glial and fibrous scars helps to contain the damage, preventing it from spreading and affecting surrounding tissue. The functions of these barriers are not fully understood yet, but their inhibitory effect on axonal growth has been extensively documented (Fawcett and Asher, 1999; Sandvig et al., 2004; Silver and Miller, 2004; Yiu and He, 2006; Fitch and Silver, 2008).

#### Inhibitory elements for axonal growth

Various elements have been described as being responsible of the adverse environment for axonal growth. The glial scar constitutes a physical barrier that impedes passage of axons across the lesion site (Figure 1A). In addition, the activated glial cells in the scar secrete ECM components, especially chondroitin sulphate proteoglycans (CSPGs) such as Neurocan, Brevican, Versican and NG2 (Fawcett and Asher, 1999; Tang et al., 2003), which exert an inhibitory influence on axonal growth (Shearer and Fawcett, 2001; Tang et al., 2003; Silver and Miller, 2004). Activation of Rho small GTPase proteins which are recognized by CSPGs blocks actin polymerization in growing neurites (Sandvig et al., 2004; Díaz-Martínez and Velasco, 2009). Some of the inhibitory molecules over-expressed in the site of lesion are Myelin-associated glycoprotein, Oligodendrocyte-myelin glycoprotein, Nogo protein and its receptor Nogo-66, Semaphorin 4D, Ephrin B3 (Kawano et al., 2012), Semaphorin 3D (Pasterkamp et al., 1999) and Ephrin B2 (Bundesen et al., 2003). The lesion site has been shown to secrete chemo-repulsive molecules like Tenascin (McKeon et al., 1991) and Semaphorin 3A (Pasterkamp et al., 1999). The fibrotic scar also produces inhibitory elements: it has been reported that fibroblasts express NG2 proteoglycan (Tang et al., 2003), Phosphocan (Tang et al., 2003), Tenascin-C (Tang et al., 2003), Semaphorin 3A (Pasterkamp et al., 1999) and Ephrin B2 (Bundesen et al., 2003). These data indicate that glial and fibrous scars contribute to the low rate of axonal regeneration. One option to bypass these inhibitory effects would be to prevent scar formation, but this could imply secondary effects, such as spreading of the damage. Alternatively, a modification of the lesion environment by introducing a device that is a permissive for axonal growth is feasible.

### PNS

**Figure 2:**
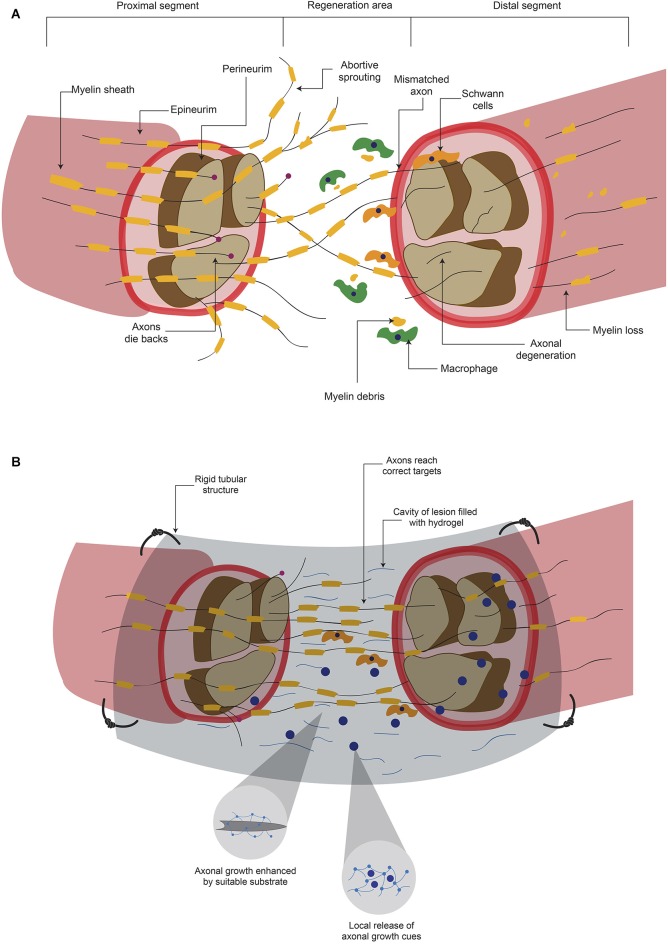
**Hydrogels promotes axonal regeneration after a peripheral nerve lesion. (A)** After a lesion where peripheral nerves are severed, inhibitory elements for axonal regeneration arise either in proximal or distal segments. Although there can be regeneration to unite both stumps, it is common that mismatches are formed. **(B)** When the lesion area is connected with a rigid tubular structure and this is filled with a hydrogel, there is a mechanical support and a suitable substrate for axonal growth. In addition, the hydrogel can serve as a carrier of molecules that promote axonal regeneration and ultimately functional recovery.

The damage produce diverse signals that indicate the neuronal cell to either go into regeneration processes or to undergo programmed cell death (Maripuu et al., 2012). Ca^2+^ entry (Maripuu et al., 2012) and the interruption of the retrograde transport system are the initial signals of neuronal damage (Dahlin, 2008). Damage to axons also release growth factors such as Ciliary Neurotrophic Factor, Leukemia Inhibitory Factor and Interleukin-6 in the site of injury (Hanz and Fainzilber, 2006; Raivich and Makwana, 2007). After a peripheral axon is cut or crushed, the distal part of the severed axon suffers a degenerative processes (Figure 2A) termed Wallerian degeneration (Mietto et al., 2008; Freeman, 2014), that consists in the degradation of axonal organelles and proteins, and disintegration of axonal structures (Mietto et al., 2008). In order to obtain successful axon regeneration, activation and proliferation of Schwann cells (SC) are needed (Dahlin, 2008). SC and macrophages phagocyte the disrupted myelin sheath and cell debris (Figure 2A) to clear the zone (Johnson et al., 2005). In addition, SC have been shown to provide a favorable substrate for regenerating axons (Maripuu et al., 2012). As a part of the regenerative processes, several axon growth cones emerge from the proximal stump (Figure 2A) and their growth is guided by Bugner bands, which are SC apposed around the basement membrane (Valls-Sole et al., 2011). Basement membrane is constituted by ECM proteins such as Laminin and contributes to the adhesion and guidance of cells during development (Silver and Miller, 2004). These emerging sprouts grow to reach the distal stump across the site of lesion. However, because the adverse environment, most of these developments disperse in various directions and become abortive (Johnson et al., 2005; Valls-Sole et al., 2011; Figure 2A). The few sprouts that successfully reach the distal stump grow in close apposition with SC (Johnson et al., 2005). To complete this processes, the regenerated axon is myelinated by SC to produce junctional nodes of Ranvier and internodal Schmidt-Lanterman incisures (Johnson et al., 2005). After the regeneration process, SC release Nerve Growth Factor (NGF) and Glial cell-Derived Neurotrophic Factor (GDNF) in the regenerated site (Maripuu et al., 2012).

#### Elements involved in the axonal regeneration process in the clinic

Although the PNS has some regeneration capacity, functional restoration is not always achieved (Hill et al., 2011; Valls-Sole et al., 2011). Factors involved in the recovery are age (Lundborg and Rosén, 2001; Ruijs et al., 2005), the period of time between injury and medical assistance (Ruijs et al., 2005), the type of nerve that is damaged and the magnitude of injury (Lundborg, 2004); for example, when the lesion consists in a moderate compression, the chances of regeneration are higher (Lundborg, 2004) than when the axon has been transected (Maripuu et al., 2012). Current treatments trying to reconnect the severed nerve offer a low success rate. One major problem in these surgical procedures is that axons do not innervate the correct area, since many axons compete to connect and this lead to loss of selectivity (Valls-Sole et al., 2011). These mismatches could convey disturbances in signaling between CNS neurons and PNS neurons, which result in sensorimotor alterations (Valls-Sole et al., 2011). Although PNS has more permissive environment in contrast to CNS, the majority of regeneration processes do not reach to innervate the pre-lesional area (Figure 2A), which leads to several adverse consequences.

## Scaffolds that promote and guide axonal growth

### Biomaterials

Biomaterials possess properties that make them biocompatible, meaning that they do not produce cytotoxic or immunologic reactions; their components should be susceptible to modifications, and the process of its production ideally should be easy and reliable (Holmes, 2002). It is important to mention that the specific requirements will dictate the origin of biomaterials. Natural biomaterials such as collagen matrix mimic largely the extracellular environment. However, natural materials present disadvantages such as lot variability (Zhang et al., 2005b), the generation of immune reactions and they may contain pathogenic agents (Holmes, 2002). On the other hand, synthetic materials can have a more consistent quality control but are not always compatible with host tissue (Metcalfe and Ferguson, 2007). Other advantages of synthetic materials are that they are free of pathogenic agents (Holmes, 2002) and could be readily modified in order to elicit a tissue response, for example, it is possible to add the isoleucine-lysine-valine-alanine-valine (IKVAV) motif which enhances cellular adhesion (Cheng et al., 2013).

### Scaffolds

The purpose of implanting a biomaterial in a tissue is to provide suitable physical support to cells in order to achieve regeneration. This substrate should mimic as much as possible the natural matrix, so it should constitute a three-dimensional (3D) structure with porous size that allows the exchange of nutrients and oxygen (Hollister, 2005; Zhang et al., 2005b). Furthermore, it needs to provide a substrate for cellular adhesion, and in selected cases, could promote guided growth, proliferation, differentiation or apoptosis by scaffold-cell or by cell-cell interactions (Hollister, 2005; Zhang et al., 2005a). Some scaffolds such as hydrogels, as we will see later, have the ability to deliver several components. Therefore, the scaffold is a dynamic element that might play an important role in the regenerative process.

### Early attempts to promote axonal regenerations using scaffolds

The first studies, aimed to promote axonal growth after injury, were performed in the PNS using rigid materials. These attempts mainly used tubular structures to protect and guide the growth of regenerating axons. However, the used materials were not biocompatible, such as silicone conduits (Cheng and Lin, 2004), mini guide channels of a polyacrylonitrile:polyvinylchloride co-polymer (Bamber et al., 1999), polytetrafluoroethylene and collagen conduits (Vasconcelos and Gay-Escoda, 2000), guidance channels of polyvinylidene fluoride (Aebischer et al., 1987), cylinders made of poly (D,L-lactic-co-glycolic acid) (Gautier et al., 1998) and poly (D,L-lactic acid) macroporous guidance scaffolds (Patist et al., 2004). Although in some cases a partial axonal regeneration was achieved, most of them did not induce successful regeneration, even after addition of trophic factors. These materials did not possess the required properties to support cell attachment, axonal growth, and some of them even induced an immune reaction. Although many biomaterials, natural or synthetic, have been proved to posses some of these properties, currently there is not a consistent strategy to induce a complete axonal regeneration either in the CNS or in the PNS.

## Synthesis and degradation of hydrogels

### Polymerization

Hydrogels are hydrophilic polymers that can incorporate up to 90% of its dry weight of water in its structure (Aurand et al., 2012; Hoffman, 2012). Water incorporation occurs during the gelling process, in which a liquid polymer solution turns into a gel structure, by polymerization of monomers (Aurand et al., 2012). Gelling involves the formation of cross-links in response to different stimuli (Sawheny et al., 1993; Aurand et al., 2012). The density of a gel can be modified, and the change in stiffness or porosity impact on the interaction of gels with cells (Lee and Mooney, 2001; Aurand et al., 2012; Kirschner and Anseth, 2013).

Several stimuli trigger the polymerization of hydrogels: temperature (He et al., 2000; Jeong et al., 2000; Tate et al., 2001), pH (Srividya et al., 2001; Cheng et al., 2009; Chiu et al., 2009), UV light exposure (Sawheny et al., 1993; Mellott et al., 2001; Bryant and Anseth, 2002; Chatterjee et al., 2010), or ionic concentration (Ellis-Behnke et al., 2006; Nagai et al., 2006; Koutsopoulos and Zhang, 2012, 2013). The formation of gels *in situ* in living tissue limits the use of UV light, extreme pH or non-physiological temperatures. Therefore, many hydrogels have been devised to initiate the gelling process when in contact with physiological temperature (Jeong et al., 2000; Tate et al., 2001), ionic concentration (Ellis-Behnke et al., 2006; Nagai et al., 2006; Koutsopoulos and Zhang, 2012, 2013) or pH (Srividya et al., 2001; Chiu et al., 2009). The *in situ* gelling process of hydrogels is unique, because the resulting polymer can take the form of the receiving tissue. This is particularly important for some lesions of the NS, in which an irregular cavity is formed and this discontinuity impedes axonal regeneration as described previously. Hydrogels can fill completely the space, whereas pre-formed structures are not suitable for this application (Macaya and Spector, 2012).

### Degradation

Degradation of hydrogels occurs by breaking of covalent bonds (Aurand et al., 2012). Several factors influence the rate of degradation. Water access is one of them: it has been shown that when hydrogels are exposed to *in vitro* conditions, hydrolysis is the main reason of bond disruption due to high availability of water; in contrast, when hydrogels are in *in vivo* conditions, enzymatic activity, in particular metalloproteases, is the principal cause of degradation (Lutolf et al., 2003; Patterson and Hubbell, 2010). Properties of hydrogel also influence this process: in high-bond density hydrogels the disruption begins from the surface, in contrast to low-bond density hydrogel, where it begins from the interior of the structure, due to the ability of water or enzymes to penetrate the hydrogel (von Burkersroda et al., 2002).

### Hydrogels as delivery systems

Hydrogels have properties that could make them a good alternative as a drug release system. During the gelling process it is possible to incorporate different types of molecules or cells into the gel structure (Nagai et al., 2006; Kobsa and Saltzman, 2008; Censi et al., 2012; Koutsopoulos and Zhang, 2012). The incorporation of molecules into hydrogel is facilitated by the high quantity of water that permits the uptake and diffusion of soluble molecules (Nagai et al., 2006; Censi et al., 2012; Koutsopoulos and Zhang, 2012). The incorporation and release process is dictated by the characteristics of the hydrogel such as the size of porous and the molecular properties such as the monomer’s molecular weight and its electrical charge (Nagai et al., 2006; Censi et al., 2012). In the case where the porous size is bigger than the molecule, the release occurs by diffusion (Amsden, 1998). On the other hand, when the molecule is larger than the porous, degradation, swelling and erosion of hydrogel permit the delivery of the molecule (Censi et al., 2012).

Administration of drugs is a necessity in the treatment of many injuries and diseases; however, commonly these drugs inside the body are metabolized and therefore have a limited time window to exert their actions. A local and controlled delivery of drugs could improve the treatments of many diseases or injuries, especially those that occur in the CNS. The delivery of drugs in the CNS implies a great challenge because the presence of BBB and the blood–spinal cord barrier, that impede the passage of many substances to the CNS (Pakulska et al., 2012). Some current delivery drugs methods into the CNS are bolus injection and catheter/minipump systems (Pakulska et al., 2012). Bolus injection into the intrathecal space is affected by the constant flow of cerebrospinal liquid, which disperse the drug, reducing its local effect (Pakulska et al., 2012). On the other hand, the use of a catheter/minipump system has high infection probabilities, due to the external minipump location. Furthermore, it is frequent that catheters suffer dislodgement, kinking, tearing and disconnection (Penn et al., 1995).

Because of the *in situ* gelling process, it is possible to use hydrogels as a local delivery system (Censi et al., 2012; Koutsopoulos and Zhang, 2012; Macaya and Spector, 2012; Pakulska et al., 2012), although it will be necessary to find out the best alternative to introduce the hydrogel into the brain or another site of the NS. It is feasible to put the hydrogel in a damaged area and release molecules there, allowing the local delivery of a drug, which could enhance the effectiveness of treatment. In addition, the use of biodegradable hydrogels is especially relevant for long-term treatments, since it will prevent repetitive invasive interventions. The data demonstrate that hydrogels are a very versatile release system because is possible to manipulate the rate of delivery and the rate of degradation. Hydrogels could be modified to release some medicament depending on the specific circumstances, like the half-life of the medicament, the dosage or the time that is required for treatment.

## Hydrogels promote axonal regeneration

Hydrogels present characteristics that make them good candidates to fulfill the needs required to promote axonal regeneration after lesions of the CNS, such as filling up the cavity of a lesion with a suitable substrate (Figure 1B). Although the PNS environment is less restrictive for axonal growth, as described previously, there are many challenges to achieve a successful regeneration. It is essential to reduce the probability of mismatches by providing guidance cues for correct reinnervation, and hydrogels can help to attain this task (Figure 2B). However, a note of caution is appropriate because, in addition to act as scaffold and delivery tools, hydrogels might represent a physical barrier for both cellular and axonal reorganization. In this section we review the published evidence showing that hydrogels promote axonal regeneration both *in vitro* and *in vivo*.

### *In vitro* studies

Hydrogels properties such as its high water content, their porous constitution and the three dimensional (3D) networks formed during gelling, mimic to some extent the ECM found in tissues (Geckil et al., 2011; Aurand et al., 2012; Kirschner and Anseth, 2013), making possible to culture cells in 3D structures *in vitro*. These structures are closer to the *in vivo* environment than the classic two-dimensional cultures (Zhang et al., 2005b).

Early work (Holmes et al., 2000) aimed to obtain self-assembling peptides with motifs similar to the arginine-glycine-aspartate (RGD) present in several ECM proteins. The authors substituted glycine with alanine (A) and repeated the RADA sequence several times. To assess the suitability of this self-assembling peptide hydrogel, a direct comparison with Matrigel (a commercial substrate containing ECM derived from carcinoma cells) was made after culturing rat hippocampal neurons. No differences in synaptic activity measured by the endocytosis marker FM1-43 were found, showing that this hydrogel can support neuronal maturation. Recently, RADA-containing peptides were used to form 3D structures to allocate neural stem progenitor cells (NSPC) in order to evaluate proliferation and neuronal differentiation (Koutsopoulos and Zhang, 2013). Both Matrigel and the self-assembled peptide-based hydrogels sustained these parameters. Matrigel was efficient during the first 2 weeks, but the hydrogel allowed neuronal survival for over 5 months. These data demonstrate that hydrogels support neuronal differentiation and long-term survival with signs of maturity.

In another study, using dopaminergic cells, Semaphorin 3A was coupled to a PEG hydrogel containing silica particles to assess the effects on axonal growth. Semaphorin 3A is a soluble protein implicated in the axonal growth of dopaminergic neurons during brain development (Hernández-Montiel et al., 2008). Application of recombinant Semaphorin 3A to these dopaminergic neurons obtained from the developing midbrain or from *in vitro* differentiated mouse embryonic stem cells caused increased growth of axons in a collagen gel system in culture. This effect was neutralized by anti-Neuropilin receptors (Tamariz et al., 2010). A significant increase in axonal length was observed with the PEG hydrogel containing either 2 or 5 µg/ml Semaphorin 3A compared to controls (Tamariz et al., 2011). Recently, a PEG hydrogel device was designed to evaluate the influence of distance in the application of a potential axonal growth-promoting molecule on murine embryonic stem cell-derived neurons. These authors conjugated Insulin-like Growth Factor 1 to poly-lactic-co-glycolic acid particles with several admixtures that resulted in different release kinetics: early, intermediate and late. The optimal conditions were: (i) a distance up to 2 mm between the poly-lactic-co-glycolic acid particles and neurons; (ii) a sequential array of early, intermediate and late release conjugates; (iii) the early release particles placed closer to the cells and those with late kinetics placed away (Lee et al., 2014).

An intermediate step between two-dimensional *in vitro* cultures and *in vivo* studies is the culture of organotypic slices, because they maintain the ECM and the 3D organization. In cultured spinal cord slices placed on different substrates such as membrane inserts, Collagen gel, soluble hyaluronic acid and hyaluronic acid-based hydrogel, different cell-type specific markers were analyzed. The hydrogel group preserved better than the other groups the characteristics of the slice: more neurons (NeuN+), a greater proportion of choline acetyltransferase-positive neurons, well-preserved astroglia and less number of activated microglial cells were reported (Schizas et al., 2014). The data of these works confirmed that hydrogels could be used as a delivery system to promote axonal growth, and preserve better the organotypic cultures. These characteristics might be useful to promote a successful axonal regeneration *in vivo*.

### *In vivo* studies

#### Hydrogels as a strategy for promoting regeneration in the brain

Several groups have assessed the biocompatibility of hydrogels in the absence of a lesion. PEG hydrogels with 13% and 20% macromer weight, and 20% PEG conjugated with GDNF were implanted into the cortex and striatum of nonhuman primates. Four months after implantation the astroglial and microglial reactions were present around the implant site of all groups, including sham. The 13% PEG hydrogel generated fewer reactions, probably due to its faster degradation (Bjugstad et al., 2008). This same group evaluated the biocompatibility of different weight percent of PEG hydrogel implanted as strands across the rat brain. The analysis in striatum revealed that both 13% and 20% hydrogels attenuate the acute response of reactive glia, compared to the sham group after 56 days (Bjugstad et al., 2010). However, when PEG was conjugated with silica particles and implanted into striatum, a higher amount of macrophages and glial cells were founded around the injection site after 30 days, compared to controls. The authors correlated this enhanced glial reaction with the presence of silica particles that were not degraded (Tamariz et al., 2011).

In some cases, one important limitation for axonal regeneration is the presence of a cavity in the damaged tissue (Figure 1A). Hydrogels have been proved to be able to fill such cavity and promote axonal growth (Figure 1B). After resection of a fraction of the cerebral cortex, the resulting cavity was filled with a hydrogel based on self-assembling peptides or with saline solution. After 6 weeks the hydrogel significantly reduced the lesion volume, and cellular ingrowth was detected. A significant decrease in astrocytic cells and macrophages was observed in the first 2 weeks, compared to the saline group (Guo et al., 2009). Hou et al. also caused a cortical damage in rats, but they used hyaluronic acid-based hydrogel, either alone or modified to incorporate Laminin in its structure, to fill the cavity. After six and twelve weeks hydrogels allow cell infiltration, angiogenesis and inhibition of the glial scar; however, only the hydrogel with Laminin was permissive for neurite growth (Hou et al., 2005).

One of the first attempts to evaluate the implantation of hydrogels together with living cells in the brain was made by Woerly et al. (1996). In this study SC, neonatal astrocytes or cells dissociated from embryonic cerebral hemispheres were entrapped in (N-(2-hydroxypropyl)-methacrylamide)-based hydrogel. Hydrogels containing SC were implanted into the rat neocortex, and promoted cellular and axonal ingrowth within the polymer. In another study, NSPC were encapsulated into self-assembling peptide hydrogels modified to include the IKVAV motif derived from Laminin (Cheng et al., 2013). Such cell-containing hydrogels were used to fill the cavity caused by a mechanical lesion in the neocortex of rats. Hydrogels made with the IKVAV motif and NSPC promoted better tissue regeneration and presented neurogenesis, compared to hydrogels without the motif, which promoted modest tissue regeneration and had prevalent glial differentiation. This study is in agreement with previous data (Hou et al., 2005) that demonstrate that the Laminin or Laminin-derived motif incorporated to hydrogel structure allow the recovery of tissue continuity. Another group tried to promote tissue recovery with a different strategy, which consisted in incorporating GDNF to gelatin-based hydrogels, with the objective to attract to the site of lesion the endogenous NSPC present in the adult subventricular zone. The hydrogel loaded with GDNF attenuated the astroglial reaction, promoted neurite growth into the site of lesion and induced the migration of neuroblasts towards the lesion site. However, cells did not reach the site of lesion, and the migration effect was observed only at 7 days post-lesion, disappearing after 21 days (Fon et al., 2014).

Probably the most important aspect in the CNS is to achieve re-connection of damaged areas, which in the long run might positively impact behavior. In a model where hamsters’ optic tracts were severed and the resulting cavity was filled with a self-assembling peptide hydrogel or with saline solution, researchers observed that the hydrogel helped to reconnect the areas around the lesion after 6 weeks, in contrast to saline-treated animals. More importantly, vision was improved in the hydrogel-treated group (Ellis-Behnke et al., 2006). Although the degree of axonal re-growth varies depending on the strategy used, hydrogels have been demonstrated to be able to fill the lesion cavity with a suitable substrate for axonal growth, and the further addition of trophic factors or cells increases the possibilities of improvement after traumatic lesions in the brain (Figure 1B). However, in general terms, the evidence is still insufficient to say that hydrogels would substitute current treatments for brain lesions.

#### Hydrogels promote regeneration in the spinal cord

The studies showing that hydrogels promote axonal growth *in vitro* prompted investigators to test if implantation of hydrogels into the damaged spinal cord could promote recovery. A hyaluronic acid hydrogel was evaluated *in vitro* and *in vivo* to investigate if it was able to promote neurite growth. Although this hydrogel promoted neurite growth *in vitro*, it was not sufficient to achieve functional recovery when implanted in rats with complete transected thoracic spinal cord (Horn et al., 2007). Transection of the spinal cord in cats followed by filling of the cavity with NeuroGel hydrogel (N-(2-hydroxypropyl) methacrylamide) permitted the formation of neural tissue with myelinated axons across the damaged area, connecting both sides of the cavity, and allowing infiltration of glial cells and capillary vessels (Woerly et al., 2001). In additional work, the authors found that the hydrogel prevented scar formation and that the gliosis reaction was reduced in the interface between tissue and hydrogel (Woerly et al., 2004).

One possibility in the design of a suitable scaffold is the combination of two different types of hydrogels, to obtain a better substrate. A combined poly lactic acid (PLA) and poly (2-hydroxyethyl methacrylate, PHEMA) hydrogel was devised to obtain a degradable porous structure. This mixed hydrogel was implanted in the hemisected spinal cord of rats and demonstrated to promote axonal growth into the lesion area; moreover, animals improved in the widely used behavioral Basso, Beattie and Bresnahan scale (Pertici et al., 2014). Another strategy evaluated to bridge the two stumps after a spinal cord lesion is the use of tubular structures, which provide mechanical support for axonal regeneration. A tubular device made with poly (2-hydroxyethyl methacrylate-co-methyl methacrylate, PHEMA-MMA) hydrogel was used to join the transected spinal cord of rats and this strategy resulted in the re-establishment of tissue continuity, allowing axon regeneration with minimal scar tissue. Although the empty tubular structure promoted recovery by itself, it was proposed that filling the tubular structure with a suitable substrate could be a better option to promote recovery (Tsai et al., 2004). The same group evaluated this approach (Tsai et al., 2006), filling the tubular structure used previously with matrices of Collagen, Fibrin, Matrigel, methylcellulose or smaller PHEMA-MMA tubes. In addition, Fibrin and Collagen were supplemented with Fibroblast growth factor-1 (FGF-1) and NT-3. It was observed that almost all the matrices used promoted more axonal regeneration compared to unfilled structures. The addition of FGF-1 increased the axonal regeneration of vestibular neurons, and the addition of NT-3 decreased the total number of axons regenerating from brainstem neurons.

In addition to promote axonal regeneration, hydrogels can incorporate into its structure trophic factors and release them on site after a spinal cord lesion. PHEMA hydrogels soaked with BDNF and control PHEMA hydrogels were implanted into the hemisected spinal cords of rats. Only the BDNF-containing hydrogel allowed axonal growth into the polymer structure (Bakshi et al., 2004). Similarly, BDNF was embedded into agarose hydrogel and implanted into hemisected spinal cords of rats. It was demonstrated that it promoted greater axonal growth in contrast to hydrogels without BDNF (Jain et al., 2006). Another trophic factor evaluated in spinal cord lesions is NT-3, which was combined to a hydrogel of acrylated PLA-b-PEG-b-PLA to release it in hemisected cord of rats. Animals treated with hydrogel and NT-3 presented more axonal growth into the lesion site and improved in the behavioral parameters, in contrast to animals treated only with hydrogel (Piantino et al., 2006). Collectively, these experiments strongly suggest that incorporation of molecules that promote axonal growth and/or cell survival increases the possibilities of recuperation after a spinal cord lesion.

As mentioned earlier, hydrogels can incorporate living cells, making grafting of cells together with hydrogel an additional strategy to promote recovery. SC and NSPC have been implanted with and self-assembling peptide-based hydrogel into transected spinal cord of rats. The ingrowth of tissue to the lesion was better in animals treated with hydrogel-embedded cells than those treated with hydrogel alone. NSPC and SC can survive, migrate, and differentiate into the site of lesion, with SC promoting greater axonal growth into damaged area (Guo et al., 2007). Another study demonstrated that when the same hydrogel was implanted with SC in a moderate spinal cord contusion model in rats, there was a reduction of astrogliosis reaction, a motor recovery and infiltration of endogenous SC to the lesion site was observed (Moradi et al., 2012).

#### Hydrogels promote regeneration in the PNS

Although the PNS allows some degree of regeneration, mismatches are frequent and limit its recovery. The best strategy to promote axonal regeneration in peripheral nerve injuries is the use of autografts, but this convey some problems such as donor tissue morbidity and loss of function in the tissue innervated by donor nerve (Schlosshauer et al., 2006). Some attempts have been made to substitute the autografts with variable results. Hydrogel porous tubes constructed with PHEMA-MMA were implanted into interrupted sciatic nerves. At early times the autografts were more effective as evaluated by histomorphology and electrophysiology. However, after 8 weeks the scaffold showed a bimodal recovery: 60% of animals surpassed the autografts but rest did not, probably due to tube collapse (Belkas et al., 2005). A more rigid hybrid conduit was designed with poly (3,4-ethylenedioxythiophene, PEDOT) and agarose hydrogel. This device was implanted in 10 mm peroneal nerve gaps and the regeneration was evaluated by muscle mass, contractile force measurements and nerve histomorphometry. The hybrid conduits promoted better regeneration compared to agarose-only hydrogel, but autografts presented much better results (Abidian et al., 2012).

Filling of conduits with a substrate, which allows axonal growth, could enhance the regenerative process (Figure 2B). An empty blood vessel filled with a self-assembling peptide hydrogel, implanted in a sciatic nerve gap of 10 mm, promoted higher numbers of growing and re-myelinated axons, more SC infiltration, less presence of lymphocytes and macrophages, greater gastrocnemius muscle recovery and better behavioral improvement, compared to empty conduits. However, the recovery was not comparable in retrograde labeling and electrophysiology, to unlesioned animals (Zhan et al., 2013). Another group developed a Keratin-based hydrogel to fill commercial tubular conduits, which improved histological characteristics such as number of blood vessels, axons per area, and axon size. Furthermore, electrophysiological features such as conduction delay and impulse amplitude were better than with the empty tubular structures, and comparable to autografts. These results were obtained in mice with a 4 mm gap in the tibial nerve (Sierpinski et al., 2008) and in rabbits with a 2–3 cm sciatic nerve break (Hill et al., 2011). The same group characterized the early cellular response after implantation of a commercial tubular structure filled with Keratin-based hydrogel, Matrigel or saline solution in rats presenting a 1 cm sciatic nerve injury. Significant differences present in the hydrogel group compared to others were: an earlier migration of dedifferentiated endogenous SC from the proximal end, faster SC dedifferentiation, higher myelin debris clearance, and decreased macrophage infiltration. However, others parameters, such as axon density, SC labeling or the amount of cells in the distal nerve did not present differences (Pace et al., 2013). It is worth mentioning that this is the only study in which the cellular response was characterized post-hydrogel implantation after peripheral nerve injury.

The release of molecules *in situ* after a lesion could enhance axonal growth through the lesion, contributing to reinnervation of correct areas (Macaya and Spector, 2012), increasing the possibilities of recovery (Figure 2B). Animals suffering from a 10 mm gap in the sciatic nerve were implanted with PHEMA-MMA hydrogel porous tubes, filled with Collagen matrices supplemented with NT-3, BDNF and FGF-1. The rats treated with growth factors presented better axonal regeneration compared to animals receiving empty tubes, or Collagen without factors. Tubes filled with collagen and 10 µg/ml FGF-1 presented similar number of fibers with diameters similar to animals that received autografts (Midha et al., 2003). Similarly, polysulfone tubes filled with agarose hydrogel containing Laminin-1 and NGF, implanted in the severed sciatic nerve caused equivalent recovery to animals that received autografts in parameters such as morphology of the regenerated nerve and the density of myelinated axons. However, although the functional recoveries of sciatic nerve were similar after hydrogel or autograft treatment, these values were significantly lower than the non-lesioned condition (Yu and Bellamkonda, 2003).

Ultrafiltration membrane conduits filled with a self-assembling peptide hydrogel containing SC were implanted into the damaged sciatic nerve. This device caused better axonal growth and linear alignment of nerve fibers with SC than conduits filled with: (a) self-assembling peptide-only hydrogel; (b) alginate/Fibronectin hydrogel or (c) alginate/Fibronectin with SC. (McGrath et al., 2010). These studies demonstrated that the combination of a tubular structure, which provides mechanical support, filled with hydrogels increases the possibilities of axonal regeneration after peripheral nerve injury. In addition, this system could be improved by the addition of trophic factors or cells (Figure 2B). The reported recoveries are to some extent similar to those resulting from autografts, the current gold standard to treat peripheral nerve damage. However, further studies that evaluate the recovery with additional parameters, such as electrophysiological studies and anterograde/retrograde labeling through regenerated axons across the damaged area are still needed.

## Conclusion

Increasing the possibilities for axonal regeneration after neuronal damage is a complex challenge, because it is necessary to overcome several limitations, which might imply different strategies. Hydrogels have demonstrated to be useful to overcome some of these barriers, particularly by providing an adequate substrate for axonal growth. Their versatility allows modification of important parameters, which can positively impact on axonal regeneration, and this is a significant advantage compared to other biomaterials. Although different strategies such as implantation of hydrogel, alone or combined with trophic factors or with cells, have proved to promote axonal regeneration in different animal models, more research is needed to determine if hydrogels can be applied in the clinical setting in the future. Tissue regeneration seems to consistently occur after hydrogel application, but other parameters, particularly the electrophysiological and behavioral tests show more variable results, and these shortcomings will hopefully be resolved soon.

## Conflict of interest statement

The authors declare that the research was conducted in the absence of any commercial or financial relationships that could be construed as a potential conflict of interest.
